# Similarities and differences among half-marathon runners according to their performance level

**DOI:** 10.1371/journal.pone.0191688

**Published:** 2018-01-24

**Authors:** Ana Ogueta-Alday, Juan Carlos Morante, Josué Gómez-Molina, Juan García-López

**Affiliations:** 1 Department of Physical Education and Sports, Institute of Biomedicine (IBIOMED), Faculty of Physical Activity and Sports Sciences (FCAFD), University of León, León, Spain; 2 Department of Physical Education and Sports, Faculty of Education and Sport, University of the Basque Country, UPV/EHU, Spain; 3 High Sport Performance Centre of León (CAR-León), Spanish Council of Sports (CSD), León, Spain; University of Colorado Boulder, UNITED STATES

## Abstract

This study aimed to identify the similarities and differences among half-marathon runners in relation to their performance level. Forty-eight male runners were classified into 4 groups according to their performance level in a half-marathon (min): Group 1 (n = 11, < 70 min), Group 2 (n = 13, < 80 min), Group 3 (n = 13, < 90 min), Group 4 (n = 11, < 105 min). In two separate sessions, training-related, anthropometric, physiological, foot strike pattern and spatio-temporal variables were recorded. Significant differences (p<0.05) between groups (ES = 0.55–3.16) and correlations with performance were obtained (r = 0.34–0.92) in training-related (experience and running distance per week), anthropometric (mass, body mass index and sum of 6 skinfolds), physiological (VO_2max_, RCT and running economy), foot strike pattern and spatio-temporal variables (contact time, step rate and length). At standardized submaximal speeds (11, 13 and 15 km·h^-1^), no significant differences between groups were observed in step rate and length, neither in contact time when foot strike pattern was taken into account. In conclusion, apart from training-related, anthropometric and physiological variables, foot strike pattern and step length were the only biomechanical variables sensitive to half-marathon performance, which are essential to achieve high running speeds. However, when foot strike pattern and running speeds were controlled (submaximal test), the spatio-temporal variables were similar. This indicates that foot strike pattern and running speed are responsible for spatio-temporal differences among runners of different performance level.

## Introduction

The participation in long-distance running events has grown significantly in the last decade. In races between 5 km and the marathon, the total number of finishers in the USA in 2015 was about 17,114,800 runners [1]. The half-marathon was the favorite distance for male runners between 25 and 44 years of age, and finishers’ average time was around 123 min [1]. This indicates that not only elite runners take part in these events, but so do amateur runners. It is important to understand the demands and characteristics of all types of runners (i.e. recreational, moderately-trained, highly-trained), and the scientific community is interested in addressing the discipline of running from different performance-related perspectives (e.g. anthropometry, training, physiology and biomechanics).

The relationship between physiological variables and running performance has been deeply investigated. A high VO_2max_, respiratory compensation threshold and a good running economy are highly related to performance in long-distance races [2]. Some anthropometric variables are also important for good running performance, as they can affect the aforementioned physiological variables [3–5]. A lower body mass [4,5], body mass index [3,5] and sum of skinfolds [5] implies a lower muscular effort to support and accelerate the body and the legs, requiring less energy expenditure [4], lower heat production and higher heat dissipation [6], and therefore allowing a better long-distance running performance.

However, the influence of some biomechanical variables on long-distance running performance is quite unclear. Some studies identified the foot strike pattern (i.e. midfoot/forefoot *vs* rearfoot) as a key factor of performance, and found a higher percentage of midfoot/forefoot runners in the top place finishers of high-level half-marathon and marathon races [7,8]. In contrast, in low-level races this tendency was not observed [9]. On the other hand, some studies have associated a shorter contact time with better performance or running economy [7,10,11], while others have not [10,12]. These discrepancies could be due to the dependence of contact time on both running speed and foot strike pattern [13]. In regards to step rate and length, some studies observed a higher step rate in highly-trained runners compared to well-trained and non-trained ones [14,15]. This seems to be a natural adaptation to obtain an energetically more optimal step rate [10]. However, at similar running speeds, step rate and length have not been associated with performance [12].

Therefore, the main purpose of the present study was to analyze the similarities and differences between training-related, anthropometric, physiological, foot strike pattern and spatio-temporal variables in half-marathon trained runners, according to their performance level. The hypothesis was that there would be differences among runners of different level in training-related, anthropometric and physiological variables, as well as in foot strike pattern, but not in spatio-temporal variables if running speed and foot strike patterns are controlled.

## Materials and methods

### Experimental design

The study was approved by the University of León Ethics Committee. Forty-eight half-marathon runners with different performance level (from 63 to 101 min) were analyzed. Runners reported to the laboratory on two different days, with an interval of at least one week. On the first day, training-related and anthropometric characteristics were recorded, and an incremental treadmill test was performed. On the second day, a submaximal test at different running speeds was performed. The submaximal running speeds were set at 11, 13 and 15 km·h^-1^ to assure that low- and high-level runners were between 60–90% of VO_2max_ in one of these speeds, and therefore obtain their running economy [16]. During both tests, foot strike pattern, physiological (VO_2_, RER and HR) and spatio-temporal variables (i.e. contact and flight times, step rate and length) were simultaneously registered.

### Subjects

Runners were recruited from national and local track and field clubs, as well as from recreational running training groups. Finally, forty-eight long-distance male runners participated according to the following inclusion criteria: 1- runners had to be Caucasian from 20 to 50 years-old, 2- they must have participated in at least one self-selected half-marathon during the six-week period prior to the study, 3- their performance level must be better than 105 min, determined by the “chip time” (time from the start to the finish line after 21,097 m). Runners were divided into four groups according to their performance level: Group 1 (n = 11, < 70 min), Group 2 (n = 13, between 70 and < 80 min), Group 3 (n = 13, between 80 and < 90 min) and Group 4 (n = 11, between 90 and < 105 min). Additionally, following the criteria of Hasegawa et al. [8], runners were divided into two groups according to their foot strike pattern: rearfoot or midfoot/forefoot strikers, in order to 1- analyze the influence of foot strike pattern in long-distance running performance and 2- avoid the influence of foot strike pattern on spatio-temporal parameters. Written consent was obtained from the subjects and the study was approved by the University Ethics Committee.

### Procedures

All testing sessions were conducted at the same time of day (between 10 a.m. and 1 p.m.) under similar environmental conditions (~ 800 m altitude, 20–25 °C, 20–35% relative humidity). During these days, a correct intake of carbohydrate (~ 400 gr) was recommended [17]. Participants fasted for 2 h before the submaximal test and during the tests, but they were able to drink water ad libitum to avoid dehydration. Both running tests were preceded by a standardized warm-up (treadmill running at 10–12 km·h^-1^ for 10 min followed by 5 min of free stretching). All runners wore the same running shoes in every testing session (250–300 gr weight for each shoe) to prevent this variable from affecting running economy [18].

Running tests were performed on a treadmill (HP Cosmos Pulsar, HP Cosmos Sports & Medical GMBH, Nussdorf-Traunstein, Germany) with a 1% slope in an attempt to mimic the effects of air resistance on the metabolic cost of flat outdoor running [19]. Two fans with a wind speed between 4–8 km·h^-1^ (according to the preference of each runner) were placed around the treadmill (~ 50–100 cm) to cool the subjects during running [17]. Respiratory gases (Medisoft Ergocard, Medisoft Group, Sorinnes, Belgium) and heart rate (HR) (Polar Team, Polar Electro Oy, Kempele, Finland) were monitored throughout the tests. Running spatio-temporal parameters (i.e. contact and flight times, step rate and length) were recorded with a contact laser platform installed in the treadmill (SportJUMP System PRO^®^, DSD Inc., León, Spain) and connected to a specific software (Sport-Bio Running, DSD Inc., León, Spain). The spatio-temporal variables computed from this system were previously validated [20]. A minimum recording time of 20 s was set at each running speed to obtain at least 32 consecutive steps and thus reduce the effect of intra-individual step variability [13]. Runners’ foot strike pattern was determined using a high-speed video camera (240 Hz) (Casio Exilim Pro EX-F1, CASIO Europe GMBH, Norderstedt, Germany) placed on the right side of the treadmill (~ 1 m), perpendicular to the sagittal plane at a height of 40 cm from the ground. All runners were analyzed by the same observer, who identified their foot strike pattern (i.e. rearfoot or midfoot/forefoot) at their competitive running speed during the incremental treadmill test. This running speed was calculated from the time needed to complete the half-marathon (e.g. 18 km·h^-1^ for a runner with a performance of 70 min).

### Anthropometry

*S*ubject’s body mass, height and 6 skinfold measurements (triceps, subscapular, supra-iliac, abdominal, anterior thigh and medial calf) were recorded using standard equipment (HSB-BI, British Indicators LTD, West Sussex, UK). The total leg and lower leg (shank) lengths were also obtained (Harpender anthropometer, CMS instruments, London, UK), taking into account the distance from the floor to the femur (greater trochanter) and to the tibia (superior point on the lateral border of the head of the tibia), respectively. Maximal thigh and shank circumferences as well as minimum ankle circumference were measured (Holtain LTD, Crymych, UK). All measurements were made by the same researcher following the international guidelines for anthropometry [21] and the criteria of previous studies [17].

### Incremental test

The test started at 6 km·h^-1^ and treadmill speed was increased 1 km·h^-1^ every 1-min until volitional exhaustion. VO_2max_ and HR_max_ were recorded as the highest values obtained in the 30 s before exhaustion [13]. The ventilatory threshold (VT) and the respiratory compensation threshold (RCT) were identified according to the criteria of Davis [22]. Spatio-temporal parameters were recorded in the last 20 s of each running speed, from 10 km·h^-1^ (i.e. when runners started to have flight time) until peak speed [13].

### Submaximal test

Subjects performed 6-min running at 11, 13 and 15 km·h^-1^ with a 5-min rest in between. VO_2_ and HR were continuously recorded during the test, considering the average of the last 3-min period of each set as representative data [17]. Running economy was determined as the VO_2_ cost at a given running speed, expressed in ml·kg^-1^·km^-1^ and ml·kg^-0.75^·km^-1^. This last unit was chosen to avoid the possible influence of body mass in running economy [23]. The best value between 60–90% of VO_2max_ was chosen as running economy representative value [16]. Spatio-temporal parameters were recorded for a minimum of 20 s during the 5^th^ minute of each set.

### Statistical analysis

The results are expressed as mean ± SD. The Kolmogorov-Smirnov test was applied to ensure a Gaussian distribution of all results. A one-way Analysis of Variance (ANOVA) was used to analyze the differences between the four groups of runners. Additionally, the Analysis of Covariance (ANCOVA) was used to analyze the differences between the four groups of runners in biomechanical variables, taking into account as covariates runners’ foot strike pattern (i.e. midfoot/forefoot and rearfoot) and running speeds where physiological variables were obtained (i.e. peak, RCT and VT speeds). When a significant *F* value was found, the Newman-Keuls *post hoc* analysis was used to establish statistical differences between means. Effect sizes (ES) (Cohen’s d) were also calculated [20]. The magnitude of the difference was considered to be trivial (ES < 0.2), small (0.2 ≤ ES < 0.5), moderate (0.5 ≤ ES < 0.8) and large (ES ≥ 0.8). Pearson correlation coefficient (r) was used to obtain relationships between variables. SPSS+ version 17.0 statistical software (SPSS, Inc., Chicago, IL, USA) was used. Values of p<0.05 were considered statistically significant.

## Results

### Anthropometry, training-related and physiological parameters

The four groups of runners (n = 48) were not different in age (32.0 ± 7.0 years), height (176.0 ± 5.0 m), total leg length (90.0 ± 4.0 cm), lower leg (shank) length (44.0 ± 2.0 cm), and maximal thigh, shank and ankle circumferences (51.1 ± 3.1, 36.0 ± 1.0 and 22.0 ± 1.0 cm, respectively). Table 1 shows that running experience (ES = 1.62), weekly training volume (ES = 1.65), body mass (ES = 0.55), body mass index (ES = 1.42), sum of skinfolds (ES = 2.08), peak speed (ES = 3.27), VO_2max_ expressed in ml·kg^-1^·min^-1^ (ES = 1.31) and ml·kg^-0.75^·min^-1^ (ES = 1.24), speed in both VT (ES = 1.80) and RCT (ES = 3.16), and running economy expressed in ml·kg^-1^·km^-1^ (ES = 1.06) and ml·kg^-0.75^·km^-1^ (ES = 1.12) had a significant effect on performance level (p<0.01), and were related to running performance (p<0.05) (Table 1).

**Table 1:** Mean (± SD) training-related, anthropometric and physiological variables of the different groups of runners. Correlation (r) with running performance (time to complete a half-marathon).

	G1 (n = 11)	G2 (n = 13)	G3 (n = 13)	G4 (n = 11)	r
**Running performance (min)**	66.0±2.3*†#	73.0±3.4†#	85.2±2.5#	96.0±3.2	---
**Running experience (years)**	16.5±5.6*†#	11.0±3.7†#	4.5±3.3	3.6±4.2	-**0.75**
**Training volume (km·week^-1^)**	118.6±30.3*†#	85.8±23.3†#	51.7±21.3	43.3±15.4	-**0.80**
**Mass (kg)**	66.5±5.3†#	68.1±5.0†	73.0±5.6	73.0±8.9	**0.45**
**Body mass index (kg·m^-2^)**	21.4±1.4†#	21.1±0.9†#	23.3±1.3	24.1±2.4	**0.64**
**∑ of 6 skinfolds (mm)**	37.4±9.1†#	40.4±6.3†#	58.6±13.8#	70.3±15.9	**0.78**
**Peak speed (km·h^-1^)**	22.1±0.8*†#	20.6±1.0†#	18.8±0.4#	17.4±0.9	-**0.92**
**VO_2max_ (ml·kg^-1^·min^-1^)**	69.2±5.0*†#	64.4±5.7†#	56.9±4.5	55.9±6.2	-**0.76**
**VO_2max_ (ml·kg^-0.75^·min^-1^)**	197.4±13.8*†#	184.9±14.1†#	166.1±13.2	163.1±16.0	-**0.67**
**RCT speed (km·h^-1^)**	18.6±1.2*†#	17.4±1.2†#	15.5±0.8#	13.8±1.1	-**0.92**
**RCT—% VO_2max_**	87.8±4.8	90.2±3.7	87.6±5.0	84.4±5.3	-0.33
**VT speed (km·h^-1^)**	12.7±1.2*†#	11.8±1.3†#	10.2±0.5	9.8±1.3	-**0.76**
**VT—% VO_2max_**	58.9±4.5	61.1±7.1	59.7±6.4	62.7±7.4	0.11
**RE (ml·kg^-1^·km^-1^)**	196.1±18.8#	205.5±12.1	205.2±12.9	219.5±18.4	**0.39**
**RE (ml·kg^-0.75^·km^-1^)**	559.7±55.1#	590.0±35.6	600.0±41.8	640.4±52.8	**0.50**
**RER (VCO_2_·VO_2_^-1^)**	0.79±05#	0.83±0.06	0.84±0.06	0.89±0.05	**0.51**

Note: G1, G2, G3, G4, groups of runners of different performance level (< 70, < 80, < 90 and < 105 min, respectively). ∑ of 6 skinfolds, sum of six skinfolds. VO_2max_, maximun oxygen uptake. RCT, respiratory compensation threshold. VT, ventilatory threshold. RE, running economy. RER, Respiratory Exchange Ratio.

*, significant differences with Group 2.

^†^, significant differences with Group 3.

^#^, significant differences with Group 4.

r, significant correlations (p<0.05) in bold type.

### Foot strike pattern

**Fig 1:**
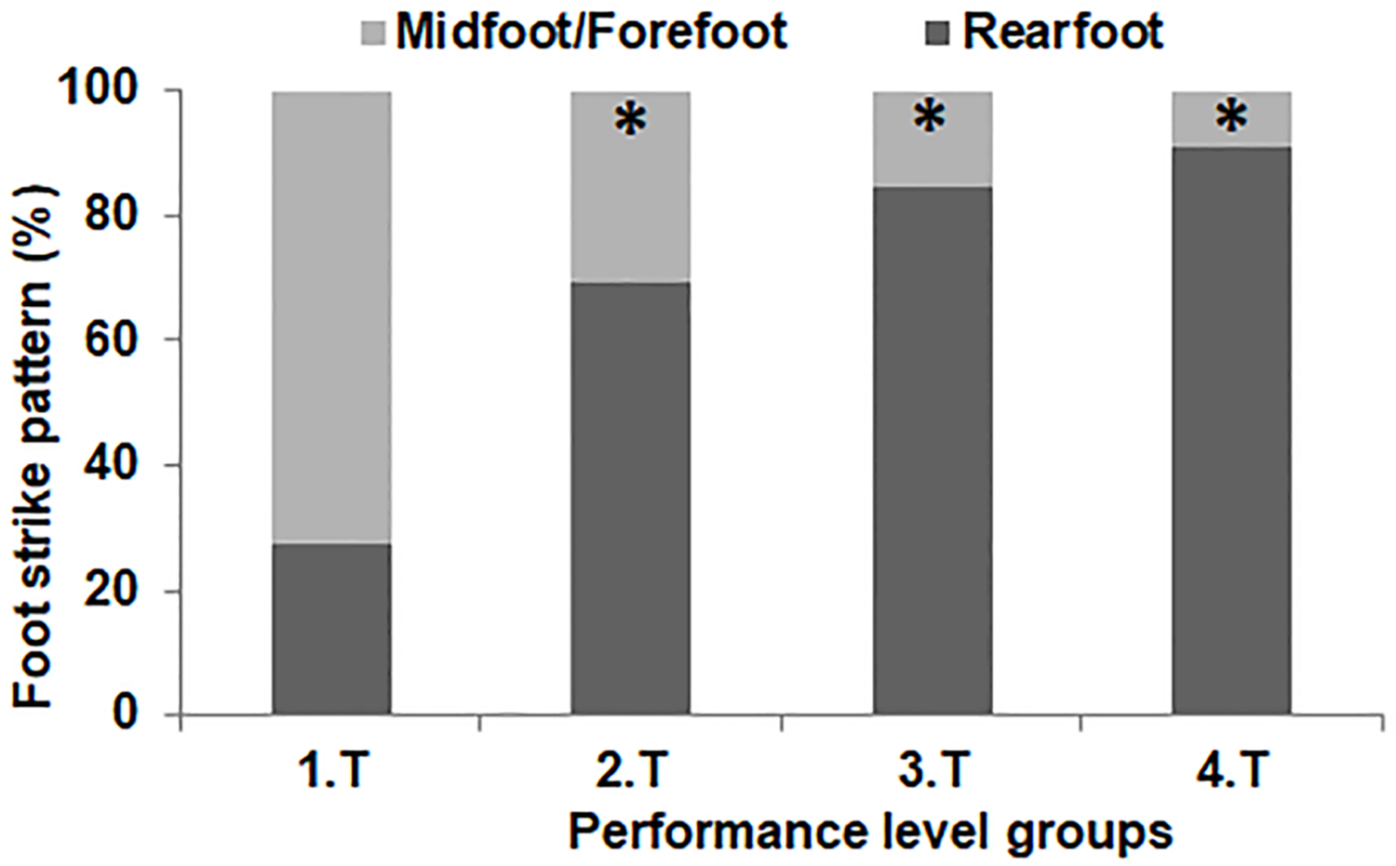
Foot strike pattern distribution (midfoot/forefoot and rearfoot) in each group of runners. G1, G2, G3, G4, groups of runners of different performance level (< 70, < 80, < 90 and < 105 min, respectively). *, significant differences with Group 1.

Fig 1 shows that performance level had a moderate effect on foot strike pattern distribution among groups (ES = 0.72, p<0.01). The percentage of midfoot/forefoot strikers was higher in Group 1 with respect to Groups 2, 3 and 4 (73, 31, 15 and 9%, respectively).

### Spatio-temporal parameters during the incremental test (comparison at the same relative physiological intensities)

Table 2 shows that, during the incremental test at different running speeds (i.e. peak, RCT and VT speeds), there were significant differences between groups of runners in contact time and step length (p<0.01), but not in step rate. Besides, significant correlations (p<0.05) between half-marathon performance (i.e. time spent) and contact time (r ≥ 0.50), step rate (r ≤ -0.38) and length (r ≤ -0.62) were observed. These differences and correlations disappeared taking into account the runners’ foot strike pattern and the running speed where these variables were obtained.

**Table 2:** Mean (± SD) spatio-temporal variables of the different groups of runners during the incremental tests. Correlation (r) with running performance (time to complete a half-marathon).

		G1 (n = 11)	G2 (n = 13)	G3 (n = 13)	G4 (n = 11)	r
**PEAK**	Contact time (ms)	177±15*†#	193±17†#	215±17	222±14	**0.76**
	Step rate (spm)	190.7±4.7	187.6±6.3	190.6±8.0	189.7±15.5	0.01
	Step length (m)	1.86±0.09†#	1.80±0.12†#	1.61±0.13	1.54±0.16	-**0.73**
**RCT**	Contact time (ms)	198±23*†#	219±19†#	241±19#	260±19	**0.82**
	Step rate (spm)	181.7±6.9	177.4±7.3	178.5±8.9	172.7±9.6	-**0.38**
	Step length (m)	1.66±0.09*†#	1.58±0.11†#	1.42±0.09#	1.29±0.10	-**0.87**
**VT**	Contact time (ms)	246±22*†#	282±34†#	304±21	313±33	**0.66**
	Step rate (spm)	167.5±4.8	166.2±8.0	162.6±6.2	159.6±6.2	-**0.43**
	Step length (m)	1.22±0.09*†#	1.13±0.12†#	1.03±0.06	1.05±0.08	-**0.62**

Note: G1, G2, G3, G4, groups of runners of different performance level (< 70, < 80, < 90 and < 105 min, respectively). PEAK, peak speed reached during the incremental test. RCT, respiratory compensation threshold. VT, ventilatory threshold. spm, steps per minute.

*, significant differences with Group 2.

^†^, significant differences with Group 3.

^#^, significant differences with Group 4.

r, significant correlations (p<0.05) in bold type.

### Spatio-temporal parameters during the submaximal test (comparison at standardized running speeds)

Table 3 shows that, at standardized submaximal speeds (11, 13 and 15 km·h^-1^), no significant differences between groups were observed in step rate and length. On the contrary, contact time was significantly shorter (p<0.01) in higher level runners (ES = 0.72, 0.74 and 0.88, respectively). These differences disappeared when the runners’ foot strike pattern was taken into account.

**Table 3:** Mean (± SD) spatio-temporal variables of the different groups of runners during the submaximal tests. Correlation (r) with running performance (time to complete a half-marathon).

		G1 (n = 11)	G2 (n = 13)	G3 (n = 13)	G4 (n = 11)	r
**11 km·h^-1^**	Contact time (ms)	258±19*†#	279±19	290±20	295±26	**0.53**
	Step rate (spm)	165.1±3.7	165.5±7.3	164.4±7.8	163.1±11.6	**0.52**
	Step length (m)	1.11±0.03	1.11±0.05	1.12±0.05	1.13±0.08	0.19
**13 km·h^-1^**	Contact time (ms)	236±16*†#	253±19	264±16	263±11	**0.51**
	Step rate (spm)	169.3±3.7	168.2±6.2	173.4±9.8	171.1±11.1	0.13
	Step length (m)	1.28±0.03	1.29±0.05	1.25±0.07	1.27±0.08	-0.10
**15 km·h^-1^**	Contact time (ms)	219±16*†#	233±16	242±15	242±11	**0.50**
	Step rate (spm)	174.9±3.6	172.1±6.6	180.5±10.3	178.5±13.0	0.23
	Step length (m)	1.43±0.03	1.46±0.06	1.39±0.08	1.41±0.10	-0.21

Note: G1, G2, G3, G4, groups of runners of different performance level (< 70, < 80, < 90 and < 105 min, respectively). spm, steps per minute.

*, significant differences with Group 2.

^†^, significant differences with Group 3.

^#^, significant differences with Group 4.

r, significant correlations (p<0.05) in bold type.

## Discussion

The main outcome of this study was that there were no differences in spatio-temporal parameters (i.e. contact time, step rate and length) among half-marathon runners of different performance level (from 63 to 101 min) when the same foot strike pattern is used and they are running at equal submaximal speed. However, high-level runners’ group exhibited the highest percentage of midfoot/forefoot strikers (~ 73%) compared to the other three groups (~ 9–31%) (Fig 1), and therefore they showed lower contact times than rearfoot strikers (i.e. low-level runners).

### Anthropometry, training-related and physiological parameters

Strong relationships between performance and training-related variables were found (Table 1). This is in line with previous studies that considered the excellence in long-distance running as the combination of genetic, environmental (i.e. socio-demographic) and training-related factors (i.e. deliberate practice theory) [24]. In the present study, in line with previous ones [3,4,5], higher level runners were lighter, had lower body mass index and lower fat/sum of skinfolds. In contrast, linear anthropometric variables (i.e. height, lengths or circumferences) had no influence on running performance, which is in agreement with some previous studies [3,4,5]. However, other studies found the contrary, which could be due to the different ethnicities compared (e.g. Caucasian *vs* African) and not to the performance level itself [17,25].

Additionally, as expected, VO_2max_, peak speed, and speed in both VT and RCT were strongly related to half-marathon performance (Table 1), in the same line with previous findings [2,11,16,26,27]. It is noteworthy the weak relationship between performance and running economy (r ≤ 0.50), coinciding with studies that did not observe any influence of this variable [12, 28]. This could be because: 1- running economy is just one factor explaining performance and it can be compensated by other factors [28]; 2- both economical and uneconomical runners have been identified at all levels of performance [29]; 3- the dependence of running economy on training status [2], as all runners in this study were well-trained; and 4- the higher percentage of midfoot/forefoot strikers in the Group 1 (Fig 1), being less economical runners than rearfoot strikers [13,30].

### Foot strike pattern

Foot strike pattern distribution among groups found in this study is in line with previous studies that compared the foot strike patterns of top and bottom place finishers of high-level half-marathon and marathon races [7,8]. Runners with a higher performance level tend to more frequently use a midfoot/forefoot strike pattern, which allows them to shorten contact time by 10% at the same running speed than rearfoot strikers [7,13,20,30–32]. This could be beneficial to reach high running speeds during training and competition (> 20 km·h^-1^) without compromising step rate [13,32]. Table 1 showed that peak running speed in Groups 1 and 2 was higher than 20 km·h^-1^, and contact time was lower than 200 ms (10% shorter than in the Groups 3 and 4), which highlights the importance of foot strike pattern to shorten contact time to achieve those high running speeds.

### Spatio-temporal parameters during the incremental test (comparison at relative physiological intensities)

The differences in spatio-temporal variables (i.e. contact time, step rate and length) among groups and the correlations with performance during the incremental test were reasonable (Table 2). All these variables are highly dependent on running speed, and as it was previously commented, contact time is also dependent on foot strike pattern. In fact, it was observed in a previous study that an increase of 2 km·h^-1^ in running speed could mean an increase of ~ 7.4 steps per minute in step rate, ~ 0.284 m in step length and a decrease of ~ 20 ms in contact time, independently of the type of foot strike pattern [13]. However, during the incremental test, when foot strike pattern and running speed were considered as covariates (i.e. ANCOVA), the differences in spatio-temporal variables disappeared. This finding suggests that foot strike pattern and running speed are responsible for spatio-temporal differences between runners.

At similar physiological intensities, step length was different among the groups of runners, while step rate was not (Table 2). This is in agreement with previous studies performed in veteran marathon runners, where shorter step length was the cause of speed reduction with age [33], possibly due to a loss of strength over the years [34]. Similarly, a strong relationship was also established between strength training and the improvement in long-distance running performance [35]. Nevertheless, to the best of our knowledge, none of these studies analyzed the effect of strength training programs on running spatio-temporal variables, which could be a future aim.

### Spatio-temporal parameters during the submaximal test (comparison at standardized running speeds)

When running speed was controlled (i.e. submaximal text, Table 3), there were no differences among groups in step rate and length, in concordance with previous findings [13,20]. On the contrary, a recent study performed in collaboration with our research group and following similar experimental procedures showed differences in both step rate and length, but not in contact time when trained and untrained runners were compared [15]. Trained runners showed higher step rate and shorter step length at the same running speeds than untrained ones. This condition (i.e. higher step rate and shorter length) could be a natural adaptive mechanism to prevent some of the most common running-related injuries as it decreases the magnitude of the center of mass vertical excursion, ground reaction force, impact shock, and may ameliorate energy absorption at the hip, knee, and ankle joints impacts during running [36]. However, when experienced runners of different performance level are compared, as the present study showed, these differences in step rate and length are not observed, probably due to the high training status of the runners (i.e. more than 40 km·week^-1^ of running, more than 3 years of running experience and a RCT above the 84% of VO_2max_) regardless their performance level.

Thereby, from the results of the present and previous studies [13,15], the association between shorter contact times and better performance in long-distance runners [7,11] is quite questionable, because it depends on both foot strike pattern and running speed. When both variables are controlled, there are no differences in contact time among runners of different performance level. In other words, contact time seems to be very consistent among highly-trained runners of different performance level, which could constitute further investigation.

## Conclusions

The present study demonstrated that runners from different performance level differed in training-related (i.e. years of experience and weekly training volume), anthropometric (i.e. body mass, body mass index and sum of skinfolds), physiological (i.e. VO_2max_, RCT and running economy), foot strike pattern and spatio-temporal variables (i.e. contact time, step rate and length). However, when foot strike pattern and running speed were controlled (i.e. running at the same absolute speed), spatio-temporal variables were similar among them. Higher level participants more frequently adopt midfoot/forefoot strike patterns and they run at higher running speeds, which implies differences in spatio-temporal variables. Nonetheless, future studies should analyze why spatio-temporal variables are so consistent when running speed and foot strike pattern are similar.

## Supporting information

S1 Dataset Individual dataset of the runners. (XLS)

## References

[1] Running USA [Internet]. Running USA annual half-marathon report; c2015 [cited 2015 Aug 21]. http://www.runningusa.org/annual-reports

[2] Basset DR, Howley ET. Limiting factors for maximum oxygen uptake and determinants of endurance performance. Med Sci Sports Exerc. 2000; 32(1):70–84. 10647532 10.1097/00005768-200001000-00012

[3] Hagan RD, Upton SJ, Duncan JJ, Gettman LR. Marathon performance in relation to maximal aerobic power and training indices in female distance runners. Br J Sports Med. 1987; 21(1):3–7. 3580726 10.1136/bjsm.21.1.3 PMC1478599

[4] Knechtle B, Duff B, Welzel U, Kohler G. Body mass and circumference of upper arm are associated with race performance in ultraendurance runners in a multistage race—the Isarrun 2006. Res Q Exerc Sport. 2009; 80(2):262–8. doi: 10.1080/02701367.2009.10599561 19650392 10.1080/02701367.2009.10599561

[5] Zillmann T, Knechtle B, Rüst CA, Knechtle P, Rosemann T, Lepers R. Comparison of training and anthropometric characteristics between recreational male half-marathoners and marathoners. Chin J Physiol. 2013; 56(3):138–46. doi: 10.4077/CJP.2013.BAB105 23656215 10.4077/CJP.2013.BAB105

[6] Cheuvront SN, Haymes EM. Thermoregulation and marathon running: biological and environmental influences. Sports Med. 2001; 31(10):743–62. 11547895 10.2165/00007256-200131100-00004

[7] Hasegawa H, Yamauchi T, Kramer WJ. Foot strike patterns of runners at the 15 km point during an elite-level half marathon. J Strength Cond Res. 2007; 21(3):888–93. 17685722 10.1519/R-22096.1

[8] Kasmer ME, Liu XC, Roberts KG, Valadao JM. Foot-strike pattern and performance in a marathon. Int J Sports Physiol Perfom. 2013; 8(3):286–92. 10.1123/ijspp.8.3.286 PMC4801105 23006790

[9] Larson P, Higgins E, Kaminski J, Decker T, Preble J, Lyons D, et al Foot strike patterns of recreational and sub-elite runners in a long-distance road race. J Sports Sci. 2011; 29(15):1665–73. doi: 10.1080/02640414.2011.610347 22092253 10.1080/02640414.2011.610347

[10] Moore I. Is there an economical running technique? A review of modifiable biomechanical factors affecting running economy. Sports Med. 2016; 46(6):793–807. doi: 10.1007/s40279-016-0474-4 26816209 10.1007/s40279-016-0474-4 PMC4887549

[11] Paavolainen LM, Nummela AT, Rusko HK. Neuromuscular characteristics and muscle power as determinants of 5-km running performance. Med Sci Sports Exerc. 1999; 31(1):124–30. 9927020 10.1097/00005768-199901000-00020

[12] Støren Ø, Helgerud J, Hoff J. Running stride peak forces inversely determines running economy in elite runners. J Strength Cond Res. 2011; 25(1):117–23. doi: 10.1519/JSC.0b013e3181b62c8a 20093965 10.1519/JSC.0b013e3181b62c8a

[13] Ogueta-Alday A, Rodríguez-Marroyo JA, García-López J. Rearfoot striking runners are more economical than midfoot strikers. Med Sci Sports Exerc. 2014; 46(3):580–5. doi: 10.1249/MSS.0000000000000139 24002340 10.1249/MSS.0000000000000139

[14] Slawinski JS, Billat VL. Difference in mechanical and energy cost between highly, well, and nontrained runners. Med Sci Sports Exercise. 2004; 36(8):1440–6. 10.1249/01.mss.0000135785.68760.96 15292755

[15] Gómez-Molina J, Ogueta-Alday A, Stickley C, Cámara J, Cabrejas-Ugartondo J, García-López J. Differences in spatiotemporal parameters between trained and untrained participants. J Strength Cond Res. 2017; 31(8):2169–75. doi: 10.1519/JSC.0000000000001679 28731978 10.1519/JSC.0000000000001679

[16] Helgerud J, Støren Ø, Hoff J. Are there differences in running economy at different velocities for well-trained distance runners? Eur J Appl Physiol. 2010; 108(6):1099–105. doi: 10.1007/s00421-009-1218-z 20024579 10.1007/s00421-009-1218-z

[17] Lucia A, Esteve-Lanao J, Olivan J, Gómez-Gallego F, San Juan A, Santiago C, et al Physiological characteristics of the best Eritrean runners-exceptional running economy. Appl Physiol Nutr Metab. 2006; 31(5):530–40. doi: 10.1139/h06-029 17111007 10.1139/h06-029

[18] Franz JR, Wierzbinski CM, Kram R. Metabolic cost of running barefoot versus shod: is lighter better? Med Sci Sports Exerc. 2012; 44(8):1519–25. doi: 10.1249/MSS.0b013e3182514a88 22367745 10.1249/MSS.0b013e3182514a88

[19] Jones AM, Doust JH. A 1% treadmill grade most accurately reflects the energetic cost of outdoor running. J Sports Sci. 1996; 14(4):321–7. doi: 10.1080/02640419608727717 8887211 10.1080/02640419608727717

[20] Ogueta-Alday A, Morante JC, Rodríguez-Marroyo JA, García-López J. Validation of a new method to measure contact and flight times during treadmill running. J Strength Cond Res. 2013; 27(5):1455–62. doi: 10.1519/JSC.0b013e318269f760 22836607 10.1519/JSC.0b013e318269f760

[21] Marfell-Jones M, Olds T, Stewart A, Carter JEL. International standards for anthropometric assessment. Potchefstroom, South Africa: ISAK; 2006.

[22] Davis JA. Anaerobic threshold: a review of the concept and directions for future research. Med Sci Sports Exerc. 1985; 17(1):6–21. 3884961

[23] Svedenhag J, Sjodin B. Body-mass-modified running economy and step length in elite male middle- and long-distance runners. Int J Sports Med. 1994; 15(6):305–10. doi: 10.1055/s-2007-1021065 7822068 10.1055/s-2007-1021065

[24] Tucker R, Collins M. What makes champions? A review of the relative contribution of genes and training to sporting success. Br J Sports Med. 2012; 46(8):555–61. doi: 10.1136/bjsports-2011-090548 22535537 10.1136/bjsports-2011-090548

[25] Larsen HB, Christensen DL, Nolan T, Sondergaard H. Body dimensions, exercise capacity and physical activity level of adolescent Nandi boys in western Kenya. Ann Hum Biol. 2004; 31(2):159–73. doi: 10.1080/03014460410001663416 15204359 10.1080/03014460410001663416

[26] Tartaruga MP, Brisswalter J, Peyré-Tartaruga LA, Avila AO, Alberton CL, Coertjens M, et al The relationship between running economy and biomechanical variables in distance runners. Res Q Exerc Sport. 2012; 83(3):367–75. doi: 10.1080/02701367.2012.10599870 22978185 10.1080/02701367.2012.10599870

[27] Gómez-Molina J, Ogueta-Alday A, Camara J, Stickley C, Rodríguez-Marroyo JA, García-López J. Predictive variables of half-marathon performance for male runners. J Sports Sci Med. 2017; 16(2):187–94. 28630571 PMC5465980

[28] Mooses M, Mooses K, Haile DW, Durussel J, Kaasik P, Pitsiladis YP. Dissociation between running economy and running performance in elite Kenyan distance runners. J Sports Sci. 2015; 33(2):136–44. doi: 10.1080/02640414.2014.926384 24916998 10.1080/02640414.2014.926384

[29] Morgan DW, Bransford DR, Costill DL, Daniels JT, Howley ET, Krahenbuhl GS. Variation in the aerobic demand of running among trained and untrained subjects. Med Sci Sports Exerc. 1995; 27(3):404–9. 7752868

[30] Gruber AH, Umberger BR, Braun B, Hamill J. (2013). Economy and rate of carbohydrate oxidation during running with rearfoot or forefoot strike patterns. J Appl Physiol. 2013; 115(2):194–201. doi: 10.1152/japplphysiol.01437.2012 23681915 10.1152/japplphysiol.01437.2012

[31] Di Michele R, Merni F. The concurrent effects of strike pattern and ground-contact time on running economy. J Sci Med Sport. 2014; 17(4):414–8. doi: 10.1016/j.jsams.2013.05.012 23806876 10.1016/j.jsams.2013.05.012

[32] Hayes P, Caplan N. Foot strike patterns and ground contact times during high-calibre middle-distance races. J Sports Sci. 2012; 30(12):1275–83. doi: 10.1080/02640414.2012.707326 22857152 10.1080/02640414.2012.707326

[33] Conoboy P, Dyson R. Effect of aging on the stride pattern of veteran marathon runners. Br J Sports Med. 2006; 40(7):601–4. doi: 10.1136/bjsm.2006.026252 16687480 10.1136/bjsm.2006.026252 PMC2564302

[34] Piacentini MF, De Ioannon G, Comotto S, Spedicato A, Vernillo G, La Torre A. Concurrent strength and endurance training effects on running economy in master endurance runners. J Strength Cond Res. 2013; 27(8):2295–303. doi: 10.1519/JSC.0b013e3182794485 23207882 10.1519/JSC.0b013e3182794485

[35] Taipale RS, Mikkola J, Vesterinen V, Nummela A, Häkkinen K. Neuromuscular adaptations during combined strength and endurance training in endurance runners: maximal versus explosive strength training or a mix of both. Eur J Appl Physiol. 2013; 113(2):325–35. doi: 10.1007/s00421-012-2440-7 22711181 10.1007/s00421-012-2440-7

[36] Schubert AG, Kempf J, Heiderscheit BC. Influence of stride frequency and length in running mechanics: a systematic review. Sports Health. 2014; 6(3):210–17. doi: 10.1177/1941738113508544 24790690 10.1177/1941738113508544 PMC4000471

